# Introduction of an Electronic Clinical Decision Support Tool to Inform Prescribing for Pediatric Diarrhea in Bangladesh and Mali: Do Provider Expectations Predict Experiences?

**DOI:** 10.4269/ajtmh.21-1248

**Published:** 2022-07-13

**Authors:** Adama M. Keita, Ben J. Brintz, Ashraful I. Khan, Md. Taufiqul Islam, Zahid Hasan Khan, Youssouf Keita, Jennifer Hwang, Eric J. Nelson, Firdausi Qadri, Samba Sow, Daniel T. Leung, Melissa H. Watt

**Affiliations:** ^1^ Centre pour le Développement des Vaccins, Bamako, Mali;; ^2^ Division of Epidemiology, University of Utah School of Medicine, Salt Lake City, Utah;; ^3^ Infectious Diseases Division, International Centre for Diarrhoeal Disease Research, Dhaka, Bangladesh;; ^4^ Division of Infectious Diseases, University of Utah School of Medicine, Salt Lake City, Utah;; ^5^ Departments of Pediatrics and Environmental and Global Health, Emerging Pathogens Institute, University of Florida, Gainesville, Florida;; ^6^ Department of Population Health Sciences, University of Utah School of Medicine, Salt Lake City, Utah

## Abstract

Nonindicated antibiotics for childhood diarrhea is a major contributor to global antimicrobial resistance. Electronic clinical decision support tools (eCDSTs) may reduce unnecessary antibiotics. This study examined how providers’ expectations of an eCDST to predict diarrhea etiology compared with their experiences using the tool. Providers were enrolled from public hospitals in Bangladesh (*n* = 15) and Mali (*n* = 15), and surveys were completed at baseline and after using the eCDST. Baseline surveys assessed expectations (utility, ease of use, and threat to autonomy), and post surveys assessed experiences in the same domains. Providers’ experiences with ease of use exceeded their baseline expectations, and providers reported less experienced threat to autonomy after use, compared with baseline expectations. Providers’ expectations of threat to autonomy significantly predicted their experienced threat to autonomy. Findings suggest that an eCDST to inform antimicrobial prescribing for diarrhea is feasible and acceptable, but training should promote local ownership for sustainability.

Antimicrobial resistance (AMR) is a threat to global public health,^1^ and improper use of antibiotics for childhood diarrhea that is viral in etiology is a major contributor.^2^ In lower and middle-income countries (LMICs), etiological diagnosis is rarely made due to resource constraints, and a large number of patients with acute diarrhea (up to 70%) are prescribed antibiotics.^3^^–^^5^ Inappropriate use of antibiotics leads to unnecessary toxicity for the individual, increased costs to the patient and health system, and a proliferation of antibiotic resistance in the community. Thus, methods for guiding appropriate use of antibiotics are urgently needed.

To address this gap, our team developed a mobile phone-based electronic clinical decision support tool (eCDST), termed the Diarrheal Etiology Prediction (DEP) app, that estimates the probability that a case of childhood diarrhea is of viral etiology. The estimation uses an algorithm of patient-level data (clinical history and symptoms) and population-level parameters (prior patients, local and regional epidemiological trends, weather patterns), which was developed based on modeling of a large multicenter study of pediatric diarrhea.^6^ The algorithm was externally validated at healthcare centers in Mali and Bangladesh. It was then integrated into a mobile phone–based application, for physicians to use during the clinical encounter to support a rapid, evidence-based decision about antibiotic prescribing.

In launching the DEP app for a clinical trial (NCT04602676P), we were aware that providers may have resistance or perceive challenges in integrating the tool into their clinical practice. Previous research has identified interpersonal and systems-level factors that influence antibiotic prescribing practices, including financial incentives and patient preferences,^8,9^ and we were cognizant that use of a smartphone-based eCDST for diarrhea management may not be compatible with norms of clinical practice. In addition, prior research and theory have noted that uptake of eCDSTs can be hampered due to clinician distrust, effort expectancies, and threat to autonomy.^10^^–^^12^ The purpose of this study was to examine how providers’ expectations of an etiology-estimating eCDST compared with their experiences of using the tool, and to assess whether preintervention expectations were a meaningful predictor of experiences with the tool.

The clinical study was conducted over a 9-week period. The study protocol was approved by the institutional review boards of the University of Utah (IRB 135830); International Centre for Diarrhoeal Disease Research, Bangladesh (IRB PR# 20003); and University of Science Technical and Technologies de Bamako, Mali (2020/122/CE/FMOS/FAPH). All participants provided written informed consent before initiation of study activities.

Healthcare workers who provided clinical care for children with diarrhea were enrolled into the study starting in October 2020 in Bangladesh and January 2021 in Mali. We enrolled 15 providers from three public hospitals in different areas of Bangladesh, and 15 providers from four public hospitals in Bamako, Mali. The sample size of this study was derived from a patient within provider cluster-level sample size calculation for the clinical trial (NCT04602676P).

Before randomization, participants completed a brief baseline survey about their expectations related to an eCDST to inform diarrhea prescribing. The baseline survey included expectations in the domains of utility (14 items, e.g., “This app will help me describe my treatment decisions to my patients/parents”), ease of use (six items, e.g., “Learning to operate the app will be easy for me”), and threats to autonomy (six items, e.g., “Using the DEP app will give me less control over clinical decisions”).

After the baseline survey, we used a random number generator to randomize clinicians to the control condition (an eCDST to guide rehydration for diarrhea^3^^,^^7^) or the DEP app (the same eCDST that also included an etiology estimation). Participants were instructed to use the assigned eCDST with all pediatric patients presenting with diarrhea over a 4-week period. After 4 weeks, there was a 1-week washout period without decision support to reduce carryover effect. Thereafter, clinicians crossed over to the other arm for the next 4 weeks. The post survey, administered at the completion of the trial, assessed providers’ experiences in the same domains of utility (14 items), ease of use (15 items), and threats to autonomy (six items).

All items were scaled 0 to 3 (*strongly disagree* to *strongly agree*). For each domain, we calculated a mean score for each participant, which was an average of the domain items. Higher scores represented higher utility, higher ease of use, and higher perceived threat to autonomy. To examine whether participants’ reported expectations of the eCDST in the baseline survey were significantly different from their experiences of the eCDST in the post survey, we calculated paired *t* tests for each of the domain scores. To examine whether expectations were a significant predictor of experience, we examined three linear regression models, including the domain expectation as the predictor and the corresponding domain experience as the outcome.

The 30 providers who participated are described in Table 1. Overall, they reported positive experiences with the eCDST at post. The average perceived utility of the eCDST was high when assessing expectation at baseline and remained high when assessing experience at post (2.13 versus 2.12, *t = *–0.208, *P* = 0.84). The average perceived ease of use of the eCDST increased when comparing expectation at baseline and experience at post (1.88 versus 2.29, *t *= 4.63, *P* < 0.001). The average perceived threat to autonomy decreased when comparing expectation at baseline and experience at post (1.33 versus 1.09, *t *= 3.11, *P* < 0.01) (Table 2). In regression models, there was evidence that providers’ experienced threat to autonomy was associated with a change in the expectation of threat to autonomy (*P* = 0.047). Additionally, there was some evidence that providers’ expectation of utility was associated with a change in their experienced utility (*P* value = 0.060) (Figure 1).

**Table 1:** Description of the sample (*N* = 30)

	Total	Bangladesh (*n* = 15)	Mali (*n* = 15)
Sex			
Male	24 (80%)	13 (86.7%)	11 (73.3%)
Female	6 (20%)	2 (13.3%)	4 (26.7%)
Age (years)			
< 40	18 (60%)	6 (40.0%)	12 (80.0%)
40–50	10 (33.3%)	8 (53.3%)	2 (13.3%)
> 50	2 (6.7%)	1 (6.7%)	1 (6.7%)

**Table 2:** Expectances vs. experiences of the clinical decision support tool across domains of utility, ease of use, and threat to autonomy (*N* = 30)

	Pre (expectation)	Post (experience)	Mean difference	*t* statistic	*P*
Utility*	2.13	2.12	−0.02	−0.208	0.84
Ease of use†	1.88	2.29	0.40	4.63	< 0.001
Threat to autonomy‡	1.33	1.09	−0.24	−3.11	< 0.01

Score = mean of all items (possible range 0–3).

* High score = high expected/experienced utility.

† High score = high expected/experienced ease of use.

‡ High score = high expected/experienced threat to autonomy.

**Figure 1.**
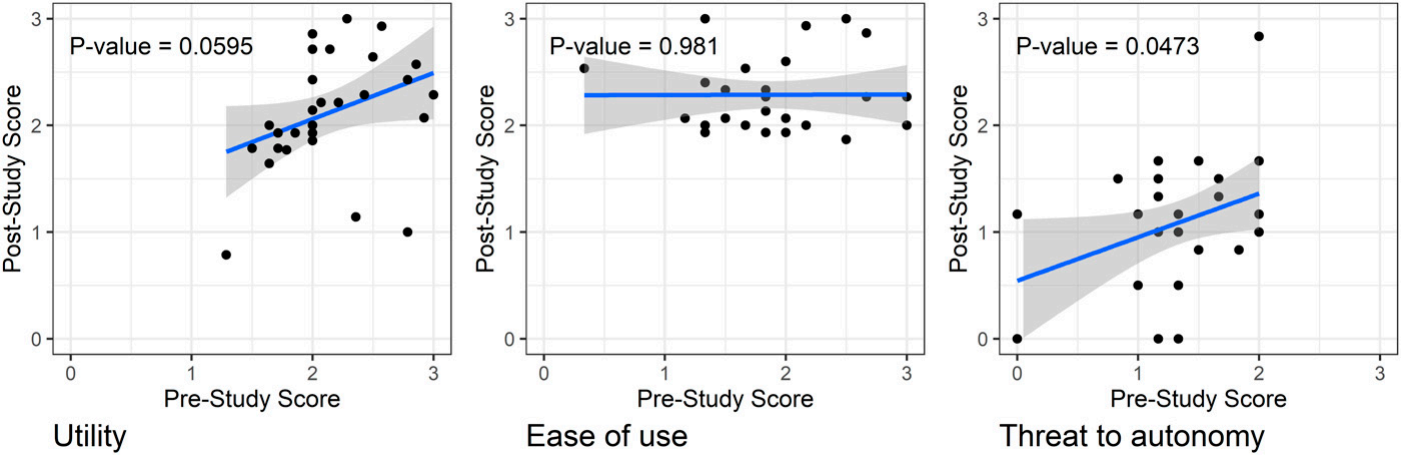
Regression models examining expectations as predictors of experiences across domains of utility, ease of use, and threat to autonomy (*N* = 30). This figure appears in color at www.ajtmh.org.

In summary, providers found the eCDST to have high feasibility (as supported by high utility and ease of use) and acceptability (as supported by low threat to autonomy). Across all domains, ratings of experiences with the eCDST exceeded baseline expectations. Providers’ expectations were a significant predictor of their reported experiences, which speaks to the need to address providers’ hesitancy about an eCDST before introduction and to facilitate local ownership and engagement throughout the process.

Our findings suggest that an eCDST to inform antimicrobial prescribing for diarrhea in LMICs is feasible and acceptable to clinical providers. The study is limited in that it was a pre–post design with a small number of participants and did not include data on how frequently the eCDST was used in clinical practice. An additional limitation is that we conducted a patient within provider cluster-level size calculation for the clinical trial; thus, this study was not powered for prespecified minimum detectable average difference between a provider’s expectations and experiences. Future research should include a larger number of clinical sites and providers and assess whether expectations predict consistent uptake of the intervention.

Clinical decision support tools that predict diarrhea etiology have the potential to reduce inappropriate antibiotic use and curb antimicrobial resistance. However, implementation of evidence-based tools may be hampered by providers’ expectancies, as they weigh the potential benefits with the potential drawbacks of using an eCDST in their clinical practice.^12^ Introducing eCDSTs into practice should include strategies to address provider expectations and harness local ownership, to promote sustainability.
